# The Natural Killer Cell Cytotoxic Function Is Modulated by HIV-1 Accessory Proteins

**DOI:** 10.3390/v3071091

**Published:** 2011-07-08

**Authors:** Bharatwaj Sowrirajan, Edward Barker

**Affiliations:** Department of Immunology and Microbiology, Rush University Medical Center, 1735 West Harrison St, Chicago, IL 60612, USA; E-Mail: bharatwaj_sowrirajan@rush.edu

**Keywords:** HIV-1, NK cells, Vpr, Vpu, Nef, NKG2D, NTB-A, PLC-γ

## Abstract

Natural killer (NK) cells’ major role in the control of viruses is to eliminate established infected cells. The capacity of NK cells to kill virus-infected cells is dependent on the interactions between ligands on the infected cell and receptors on the NK cell surface. Because of the importance of ligand-receptor interactions in modulating the NK cell cytotoxic response, HIV has developed strategies to regulate various NK cell ligands making the infected cell surprisingly refractory to NK cell lysis. This is perplexing because the HIV-1 accessory protein Vpr induces expression of ligands for the NK cell activating receptor, NKG2D. In addition, the accessory protein Nef removes the inhibitory ligands HLA-A and -B. The reason for the ineffective killing by NK cells despite the strong potential to eliminate infected cells is due to HIV-1 Vpu’s ability to down modulate the co-activation ligand, NTB-A, from the cell surface. Down modulation of NTB-A prevents efficient NK cell degranulation. This review will focus on the mechanisms through which the HIV-1 accessory proteins modulate their respective ligands, and its implication for NK cell killing of HIV-infected cells.

## 1. Natural Killer Cells Control Virus Production

An important first line of defense against viruses involves natural killer (NK) cells. Unlike cytotoxic T-lymphocytes, which also destroy virus-infected cells, NK cells respond to infected cells without prior exposure to infected cells or their components. Hence, the action of NK cells to immediately eliminate virus-infected cells and prevent the spread of the virus is essential during the early stages of viral infections since adaptive anti-viral immune responses are not fully developed. The importance of NK cells in controlling virus infections, *in vivo*, was initially shown in mice depleted of NK cells. Without NK cells, infected mice are unable to control a plethora of viruses [1–3]. Humans lacking or having dysfunctional NK cells develop fatal disseminated herpes infections [4–8]. Since NK cells are effective at eliminating virus-infected cells, many viruses that chronically infect humans utilize strategies to avoid detection and elimination by NK cells [9–12].

NK cells control viruses in one of three ways: (1) by directed release of the contents of lytic granules onto infected cells, (2) by expressing ligands (*i.e.*, Fas ligand and TRAIL) that engage death receptors on infected cells, and (3) by secreting cytokines (e.g., IFN-γ, MIP-1β) that impede or prevent virus replication [13–17]. In fact, secretion of IFN-γ by NK cells has been shown to enhance MHC class I expression on antigen presenting cells to enhance CD8+ T-cell recognition of infected cells [18]. NK cell cytotoxic granules contain perforin and granzymes. Perforin forms pores on the target cell, and granzymes enter through the pores and initiate apoptosis within the target cell [19,20]. The directed release of the contents of cytolytic granules (degranulation) is triggered by two distinct mechanisms. Antibody-dependent cellular cytotoxicity (ADCC) results in the release of perforin and granzymes when antibody-coated cells engage the low affinity receptor for IgG, CD16 (or FcγRIIIa), on NK cells [21]. Secondly, engagement of germline-encoded receptors on NK cells by their invariant ligands on the target cells regulates degranulation [22–25]. This review will focus on the latter NK cell response. In particular, we will discuss how HIV-1-infected cells manipulate surface ligands that trigger NK receptors essential in regulating degranulation.

## 2. Polarization of Lytic Granules in NK Cells Requires Stable Interactions with Target Cells

Directed degranulation by NK cells ensures that a specific, infected cell is killed leaving healthy neighboring cells unharmed [25,26]. Polarization of lytic granules towards the target cells and subsequent degranulation requires the participation of three major classes of receptors: (1) adhesion receptors, (2) activating receptors, and (3) co-activating receptors [26]. The initial step of cytolysis involves a loose association (K_d_ = ∼ 100 μM) between target cells and NK cells that require carbohydrate Ag Lewis^X^ (e.g., CD15, Gal-β1-4 GlcNAc α1-3Fuc) on target cells to interact with CD2 or selectins on NK cells [27]. Loose interactions between Ag Lewis^X^ and lectin-like receptors such as CD94 and NKG2D may allow for the NK cell to slow down and initiate longer contact with its target [28]. This loose adhesion between NK and target cells is soon followed by a stable interaction involving integrins (e.g., lymphocyte function-associated antigen (LFA)-1) on NK cells and the intercellular adhesion molecules (ICAM) family on target cells [29,30]. Once a stable interaction occurs, “firm” adhesion follows [31,32]. “Firm” adhesion involves a conformation change from a “closed” low affinity (K_d_ = ∼ 100 μM) state to an “open” configuration in which the integrins bind to ICAMs with higher affinity (K_d_ = ∼ 0.4 μM) [33,34]. The engagement of activating receptors on the NK cell with their ligands on the target cell enhances the “open” configuration state [34].

**Figure 1:**
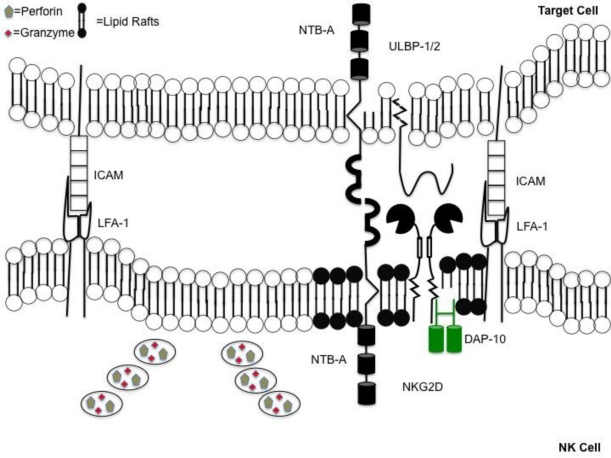
Coordinated events occur when forming an immunological synapse. Directed release of cytotoxic granules requires coordinated series of events. Synapse formation is accomplished following engagement of lymphocyte function-associated antigen (LFA)-1 on natural killer (NK) cells with intercellular adhesion molecules (ICAMs) on the target. Immunological synapse contains a “lipid raft” domain (shown in black) containing activating receptors. This is adjacent to a section of the plasma membrane allowing for the unobstructed release of perforin and granzymes, into the space between the NK cell and target.

Following the binding of integrins on NK cells to ICAMs on target cells, several events occur within the NK cells which brings the cytolytic granules towards the target cell [35]. Initially, the cytotoxic granules migrate in the direction of the microtubule-organizing center (MTOC) towards the minus ends of the microtubules found at the MTOC [36]. This is followed by actin cytoskeleton rearrangement that recruits activating receptors into lipid rafts. The clustering of activation receptors between the points of contact of the NK cell and its target forms the immunological synapse [37–39]. Following recruitment of activation receptors to lipid rafts, the MTOC containing lytic granules polarizes toward the immunological synapse due to the reorganization of the actin network [35,40]. The consequence of the MTOC containing granules polarizing to the synapse will allow for the eventual release of cytotoxic granules in an area adjacent to the activating receptors (Figure 1).

## 3. Degranulation Follows Engagement of NK Cell Activating Receptors

While integrin engagement leads to polarization of lytic granules towards the target cell, triggering integrins alone is insufficient for NK cells to degranulate [41]. “Activating” receptors (aNKRs) on NK cells being bound by their respective ligands on the target cell are required to elicit an NK cell to degranulate [42]. aNKRs lack signaling motifs in their intracellular domains. Instead, these receptors associate non-covalently with adaptor proteins through a charged residue in the activating receptor transmembrane region [24,43]. aNKRs (e.g., NKp30, NKp44, NKp46) and the killer immunoglobulin-like receptors with short cytoplasmic tails (e.g., KIR2DS1, KIR3DS1) interact with adaptor proteins containing immunoreceptor tyrosine-based activation motifs (ITAMs), defined as YXX(L/I)X_6–8_YXX(L/I) (where X_6–8_ are 6 to 8 residues between the two tyrosines) [22,44–48]. ITAM containing adaptor molecules include: DAP12, FcɛRIγ, and CD3ζ [25,49]. These adaptor proteins become phosphorylated by tyrosine kinases such as Syk and ZAP-70 allowing for NK cell cytotoxicity (See Table 1 for list of aNKRs) [50].

A second group of aNKRs does not associate with adaptor molecules containing ITAMs [24]. For example, NKG2D associates with the adaptor protein DAP10, which contains an YXXM motif. After phosphorylation, DAP10 recruits phosphatidylinositol-3-kinase (PI3K) and growth factor receptor-bound protein (Grb)-2 [51,52].

## 4. Phospholipase C-γ is the Key Mediator for NK Cell Degranulation

**Figure 2:**
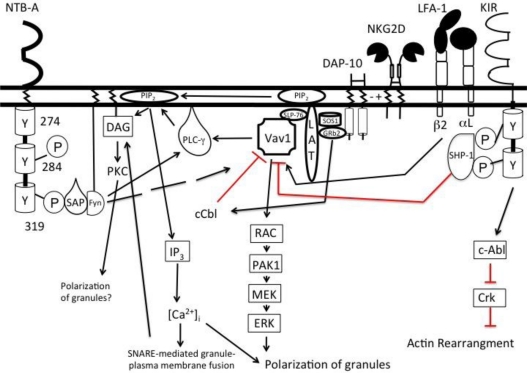
Two activating signals are required to initiate NK cell degranulation. Signals from activating (NKG2D) and co-activating receptor (NTB-A) converge to activate phospholipase-Cγ (PLCγ). Vav1, a guanine exchange factor, is able to phosphorylate PLCγ when triggered by activating receptors. However, engagement of activating receptors also phosphorylates c-Cbl, an ubiquitin ligase that negatively regulates Vav1. To overcome this inhibition, a co-activating receptor must be engaged to phosphorylate sufficient PLCγ to trigger degranulation. Degranulation is mediated by the cleavage of phosphatidylinositol into diacylglycerol (DAG) and inositol 1,4,5 triphosphate (IP_3_). DAG recruits key proteins to the plasma membrane to initiate fusion of the granule and plasma membrane. IP_3_, once cleaved, binds its receptor, IP_3_ receptor, on the membrane of the endoplasmic reticulum to open calcium channels. Ca^2+^ influx allows for the exocytosis of the cytolytic granules. Inhibitory receptors such as the KIRs can recruit phosphatases that lead to the dephosphorylation of Vav1. However, the KIRs also have the ability to activate tyrosine kinases like c-Abl leading to the inhibition of actin rearrangement.

Engagement of both adhesion and activating receptors lead to recruitment and phosphorylation of Vav1, a guanine exchange factor [53]. Vav1 ultimately regulates actin cytoskeleton rearrangement for polarization of granules and activation receptor clustering [39]. Phosphorylation of Vav1 ultimately leads to phosphorylation of phospholipase C-γ (PLCγ) following LFA-1 and/or NKG2D engagement (Figure 2). Phosphorylated PLCγ in turn hydrolyzes phosphatidylinositol 4, 5 bisphosphate (PIP_2_) to inositol 1, 4, 5 trisphosphate (IP_3_) and diacylglycerol (DAG) [54,55]. DAG recruits mammalian uncoordinated (Munc) 13-4, an essential component of the vesicle fusion complex crucial for regulated degranulation, to the plasma membrane [56]. Vesicle fusion to the plasma membrane is further facilitated by Rab27a, a Rab GTPase. Rab27a is recruited to the cell surface after LFA-1 engagement allowing for the granules to dock with Munc 13-4 at the plasma membrane [57]. IP_3_ binds to inositol trisphosphate receptor on the endoplasmic reticulum membrane and opens a calcium channel resulting in the release of calcium into the cytoplasm [58]. Calcium is necessary for the final fusion between the cytolytic granule and plasma membrane [59].

The amount of Vav1 phosphorylation induced by integrins and activating receptor(s) is insufficient to lead to degranulation. However, there may be a sufficient amount of phosphorylated Vav1 to lead to granule polarization and immunological synapse formation. This is because Vav1 is negatively regulated by the ubiquitin ligase c-Cbl. c-Cbl is induced upon engagement of an activating receptor (e.g., NKG2D), to dampen NK cell cytotoxicity, which in turn, leads to Vav1’s ubiquitination and proteosomal degradation. The loss of Vav1 decreases the levels of phosphorylated PLCγ and prevents NK cell degranulation [55]. To overcome the effects of c-Cbl’s inhibition of Vav1, NK cells require concomitant engagement of activating and co-activating receptors [55].

Co-activation receptors include the CD2 family of activating receptors (e.g., 2B4, CRACC, NTB-A) (See Table 2 for list of caNKR) [60–62]. The CD2 family of receptors associates with signaling lymphocyte activation molecule (SLAM)-associated protein (SAP) to induce cellular cytotoxicity [63–65]. When these receptors bind their respective ligands, tyrosine residues in the cytoplasmic tail become phosphorylated allowing for the recruitment of SAP. SAP binds to the tyrosine kinase, Fyn, through an arginine at position 78 in its SH2 domain [66,67]. Once bound to SAP, Fyn phosphorylates both the SLAM-family receptor as well as molecules such as PLCγ to induce the NK cell to degranulate [68]. More importantly, degranulation of resting NK cells requires at least a specific pair of activating and/or co-activating receptors to be engaged. Such pairs include NKG2D and NKp46, NKp46 and DNAM-1, and 2B4 and DNAM-1. However, combinations such as NKG2D and DNAM-1 do not result in NK cell degranulation [42].

## 5. Inhibitory Receptors Regulate Cytoskeleton Rearrangement and Synapse Formation

In addition to activation receptors, NK cell responses are regulated by inhibitory receptors (iNKRs) present on NK cells. iNKRs contain immunoreceptor tyrosine-based inhibitory motifs (ITIMs) in the cytoplasmic domain defined by the sequence (I/L/V/S)XYXX(L/V) [25]. Once the ITIMs become phosphorylated they recruit phosphatases such as Src homology region 2 domain containing phosphatase (SHP)-1 and SHP-2 [69,70]. These phosphatases lead to the dephosphorylation of signaling molecules involved in calcium influx, actin rearrangement, and granule polarization initiated by adhesion receptors [71–73]. Inhibitory receptors also activate kinases such as c-Abl which play a role in inhibiting actin rearrangement [74].

One major set of ligands to iNKRs is the major histocompatibility complex (MHC) class I molecules [45,75–77]. The MHC-I recognizing families of iNKRs are the killer-immunoglobulin (Ig)-like receptors (KIRs) with ITIM-containing cytoplasmic tails, which recognizes HLA-A, -B, and -C; the receptor pair of NKG2A and CD94, which recognizes HLA-E; and the interleukin-like transcript type 2 (ILT-2), which recognizes multiple HLA molecules. As MHC class I molecules are constitutively expressed on all healthy nucleated cells, iNKRs recognition of their MHC-I ligand inhibits the NK cell’s ability to kill healthy “self” cells. Many viruses down modulate MHC class I molecules on infected cells to avoid CD8+ T-cell responses [78]. However, MHC-I down modulation leaves the target cell susceptible to destruction by NK cells by removing inhibitory ligands [10,11]. Simply lacking or having impaired expression of MHC class I molecules is insufficient for NK cell lysis. NK cells will only degranulate and kill the infected cell after engagement of activating receptors. Moreover, NK cells are capable of degranulating target cells that have normal expression of MHC-I on its surface if activating receptors are engaged to overcome the inhibition [79].

## 6. HIV-1 Alters NK Cell Degranulation by Modulating Ligands to NK Cell Receptors

Nearly 25 years ago NK cells were found to be inefficient at killing autologous HIV-1 infected primary T-cells [80–82]. However, the mechanism to explain the inability of NK cells to lyse HIV-infected cells has only recently been discovered. Below we will discuss how HIV-1 gene products impact NK cell cytolytic function and ultimately how HIV-1 avoids destruction by NK cells.

### 6.1. HIV-1 Vpr Induces Expression of Ligands for the NK Cell Activating Receptor NKG2D

NKG2D is a type II transmembrane C-type lectin that associates with the adaptor protein DAP10 [83,84]. It is expressed on all NK cells in the blood as well as subsets of CD8+ T-cells, γδ T-cells, and NKT cells [21]. NKG2D ligands include the MHC class I polypeptide-related sequence (MIC)-A and -B, as well as the cytomegalovirus unique long 16-binding protein (ULBP)-1 thru -6 [85,86]. These ligands typically are not expressed on healthy cells; however, NKG2D ligands are expressed at relatively high levels on cells undergoing genotoxic stress [87]. DNA damage leads to NKG2D ligand expression through induction of the DNA damage sensor ataxia telangiectasia-mutated (ATM) and ataxia telangiectasia-mutated and Rad 3-related (ATR) [87]. ATM detects double stranded DNA breaks, whereas ATR detects single stranded DNA breaks, typically at replication forks [88,89]. Activation of either of these kinases will result in cell cycle arrest and subsequent induction of NKG2D ligands [90].

**Figure 3:**
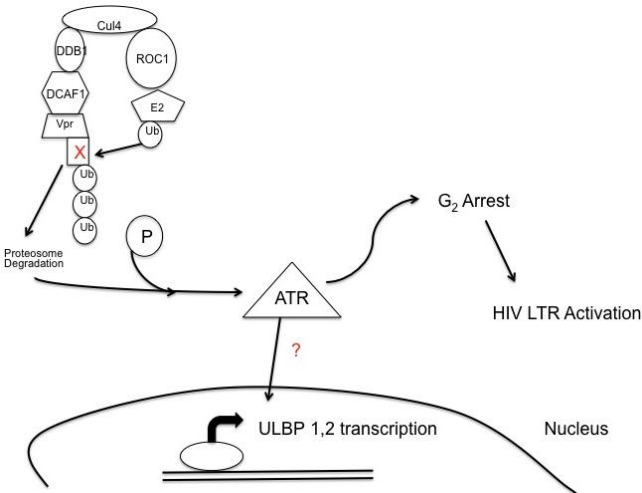
HIV-1 Vpr leads to expression of NKG2D ligands, unique long 16-binding protein (ULBP)-1 and -2. Viral protein R, Vpr, bridges DCAF1, a substrate recognition factor for an E3 ubiquitin ligase system to an unknown cell cycle protein causing for the cell cycle protein to become ubiquitinated and destroyed. The degradation of the cell cycle protein induces phosphorylation of the DNA damage sensor ataxia telangiectasia-mutated and Rad 3-related (ATR), which in turn induces expression of the NKG2D ligands, ULBP-1 and -2. Phosphorylation of ATR also leads to G_2_ cell cycle arrest creating a favorable environment for transcription of the HIV long terminal repeat (LTR).

ATR and not ATM is phosphorylated and activated during HIV-1 infection by the viral protein Vpr [88,91]. One of the benefits of ATR’s induction for HIV-1 is to induce G_2_ cell cycle arrest. The long terminal repeat (LTR) of HIV-1 is highly active during the G_2_ phase of the cell cycle resulting in higher viral production [92–94]. It has been hypothesized that G_2_ cell cycle arrest is associated with Vpr binding to DCAF1, a substrate recognition subunit for an E3 ubiquitin ligase complex, through Vpr’s leucine rich motif from residue 60–68 (Figure 3). Vpr binds not only to DCAF1 but also an unknown cell cycle protein through an arginine at residue 80 of Vpr [95–97]. The Vpr complex in turn associates with the E3 ubiqutin ligase complex, Cullin 4a/damaged DNA binding protein I (Cul4a^DDB1/DCAF1^) [98]. When the unknown cell cycle protein is ubiquitinated and destroyed by the E3 ubiquitin ligase complex, G_2_ arrest is induced and ATR becomes phosphorylated [98]. ATR’s phosphorylation through Vpr not only results in G_2_ arrest, but also leads to the expression NKG2D ligands. Vpr leads to ULBP-1 and -2 expression but not ULBP-3 through -6, or MIC-A and -B [99,100]. The specificity of NKG2D ligand expression may be due to ATR’s influence on the transcription factors Specificity Protein (SP)1 and SP3 resulting in ULBP-1 transcription [101]. Induction of ULBP-1 and -2 by Vpr provides an activating signal for NK cells to degranulate [99,100].

Nef has also been implicated in down modulating ligands for the activating receptor NKG2D [102]. Namely, it was shown that Nef down modulates MIC-A, ULBP-1, and ULBP-2. However, in more recent study, deleting Nef from HIV-1 resulted in surface expression of NKG2D ligands to the same level as wild-type infected cells [99]. Furthermore, we were never able to detect MIC-A cell surface expression on HIV-1 infected primary CD4+ T-cells. The discrepancies between the two studies have yet to be determined.

### 6.2. Nef down Modulates Ligands for Inhibitory Receptors on NK Cells

It was reported over 20 years ago that HIV-1 down modulates MHC class I molecules [103]. It was later discovered that MHC class I down modulation was due to the HIV-1 protein negative regulatory factor (Nef) [104]. In subsequent studies, Nef was found to selectively down modulate the MHC class I molecules HLA-A and -B, but not HLA-C and -E [105]. Nef is a 27 kD protein expressed early in HIV-1 infection. Nef selectively down modulates HLA-A and -B by binding to two key residues (a tyrosine at position 321 and an aspartate at position 328) in the cytoplasmic tails of HLA-A and -B. These residues are not present in the cytoplasmic tail of HLA-C preventing Nef from down modulating HLA-C [105]. While HLA-E cytoplasmic tail has the two residues found in HLA-A and -B, Nef is incapable of down modulating HLA-E. Exchanging the cytoplasmic tail of HLA-E for the cytoplasmic tail of HLA-A resulted in HLA-E’s down modulation, indicating that the cytoplasmic tail of HLA-E differs in numerous other residues to HLA-A and -B preventing Nef from down modulating HLA-E [105,106].

**Figure 4:**
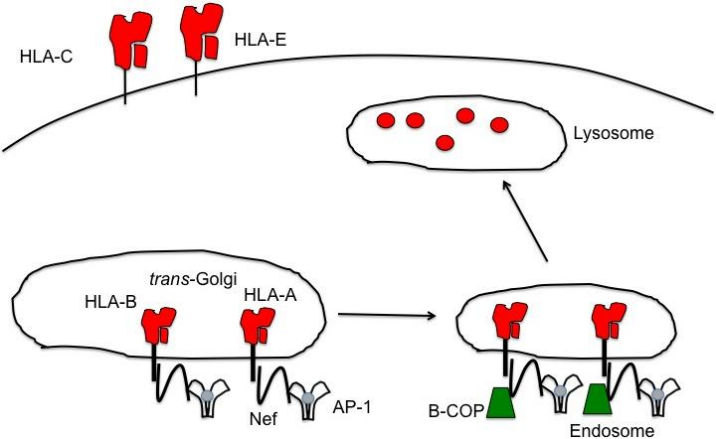
HLA-A and -B are down modulated by HIV Nef, removing an inhibitory signal for NK cells. HIV-1 Nef selectively down modulates HLA-A and -B, but leaves HLA-C and -E on the infected cell surface. Nef re-routes both HLA-A and -B from the TGN to endosomes and eventually to lysosomes for the degradation of the MHC-I molecules. Nef is able to bind to both the tail of the MHC-I molecules as well as the trafficking molecule, AP-1, to facilitate the transport of HLA-A and -B to the endosomes. Once in the endosome, Nef recruits β-COP for further transport into lysosomes. The down modulation of HLA-A and -B removes the inhibitory ligands for the NK cell inhibitory receptor KIR3DL2 and KIR3DL1, respectively. Yet, because Nef is unable to down modulate HLA-C and -E, NK cells expressing inhibitory receptors for these two MHC-I molecules will inhibit NK cell degranulation of HIV infected cells.

Upon binding to HLA-A and -B, Nef re-routes both MHC class I molecules from the *trans*-Golgi network (TGN) to endosomes by recruiting adaptor protein (AP)-1, a clathrin-mediated protein that facilitates trafficking between the TGN and endosomes [107] (Figure 4). The Nef/MHC-I complex binds to the μ subunit of AP-1 [108–110]. Nef then recruits coatomer protein-1β (β-COP), a component of COP-1 coats for transport through the early secretory pathways [111]. β-COP binds to an arginine motif (RXR) on the N-terminal of Nef [112]. Nef’s ability to bind to β-COP causes HLA-A and -B to be directed to lysosomes for their degradation. Additionally, Nef increases the internalization of MHC-I molecules from the plasma membrane through Nef’s ability to utilize PACS-1, a phosphofurin acidic cluster sorting protein [113]. PACS-1 controls endosome to TGN trafficking by binding to acidic motifs of a protein and forms a bridge to AP-1. Nef contains a cluster of glutamic acids from residue 62–65 that binds with PACS-1 connecting Nef with AP-1 [114]. In this instance, Nef forces the endocytosis of HLA-A and -B into the TGN. However, recent studies have shown that Nef primarily acts to re-route HLA-A and -B from the TGN to lysosomes, instead of promoting the internalization of MHC-I molecules via the PACS-1 mechanism [107,115,116].

It was thought that the selectivity of Nef’s down modulation of only HLA-A and -B, and not HLA-C and -E, was necessary for HIV-1 to evade responses from NK cells [105]. However, as NK cell expression of iNKRs is variegated, there are a significant number of NK cells not expressing iNKRs to HLA-C and HLA-E [117]. Because of the difference in NK cell expression of iNKRs, it stands to reason that NK cells lacking iNKRs for HLA-C and HLA-E should efficiently lyse HIV-1 infected T-cells due to Vpr’s induction of NKG2D ligands and Nef’s down modulation of HLA-A and -B. In fact, NK cells lacking iNKRs for HLA-C and -E are able to lyse HIV-1-infected cells when HLA-A and -B are down modulated [117]. These studies indicate that NK cells have the ability to kill HIV-1-infected cells. However, it should be noted that NK cell killing of HIV-1-infected cells is still two-thirds less than that observed with NK cell killing of the NK-sensitive cell line, K562 [117]. Furthermore, HIV-1 infected CD4+ T-cells have the capability to activate NK cells to the same extent as K562 cells, yet the infected T-cells induce NK cells to degranulate to a third of the level of NK cells exposed to K562 cells [118].

### 6.3. NK Cell Degranulation is Hindered by Down Modulation of NTB-A on HIV-1 Infected Cells by Vpu

Why NK cells are activated but unable to degranulate in the presence of HIV-1 infected cells was a mystery until recent work illustrating the need for not only an activating receptor but also a co-activating receptor to be engaged for NK cell degranulation [42]. NK, T-cell, B-cell antigen (NTB-A) is one such co-activating receptor that is expressed on all blood NK cells as well as CD4+ T-cells [119]. In addition, NTB-A is down modulated on infected T-cells by HIV-1 [120,121].

NTB-A is a type I transmembrane protein and a member of the signaling lymphocytic activation molecule (SLAM) receptor family [119]. It is a part of the Ig superfamily containing a distal V-type and a proximal C2-type domain in the extracellular portion of the receptor. NTB-A forms a homotypic ligand-receptor pair. When bound by its ligand, two of NTB-A’s three immunoreceptor tyrosine-based switch motifs (TXYXXV/I) are phosphorylated by tyrosine kinases such as Lck [43,122]. Phosphorylation of tyrosine 284 results in binding of Ewing’s sarcoma-activated transcript (EAT)-2, a SAP family member. EAT-2 binding to NTB-A has been implicated in cytokine production by primary NK cells [123]. Phosphorylation of tyrosine 319 results in SAP binding to NTB-A [63,124]. SAP then binds the protein kinase Fyn, which in turn, triggers NK cells degranulation [67]. While triggering NTB-A alone is insufficient to induce NK cells to release their granules, when triggered together with NKG2D, NK cells are able to degranulate [118].

Since ligand binding of NTB-A on NK cells has been shown to induce NK cell cytotoxicity, it seems likely that HIV-1-infected cells down modulate NTB-A to evade NK cells. More specifically, recent work has shown the HIV accessory protein Vpu to be both necessary and sufficient for NTB-A’s down modulation [118]. Vpu’s transmembrane region binds to NTB-A and sequesters NTB-A within the TGN [125]. Furthermore, Vpu does not increase the internalization of NTB-A and does not alter the steady-state levels of NTB-A expressed in the cell [118].

Vpu’s down modulation of NTB-A is distinct from the down modulation of CD4 and tetherin/BST-2. For both CD4 and tetherin/BST-2, Vpu serves as an adaptor to β-TrCP, a human F box protein that serves as a substrate recognition receptor for the E3 ubiquitin ligase complex. Phosphoserines at positions 52 and 56 of Vpu are necessary for the interaction between β-TrCP and Vpu. Ubiquitinized CD4 is degraded by proteasomes, whereas ubiquitinized tetherin/BST-2 is transported to acidified endosomes though this may not be the only mechanism of Vpu’s down modulation of tetherin/BST-2 [126–132]. However, this is not the case with Vpu’s down modulation of NTB-A. Mutations of the serine residues at position 52 and 56 result in down modulation of NTB-A to similar levels as wild-type Vpu. Additionally, NTB-A’s association with Vpu does not lead to its degradation by proteasomes nor is NTB-A trafficked to acidified endosomes for its destruction [118].

Thus, it is likely that NTB-A is unable to recycle back to the cell surface, removing the second activating signal needed to trigger NK cell degranulation. NTB-A’s down-modulation by Vpu prevents NK cell degranulation and lysis of HIV-1-infected T-cells by three-fold [118]. Interestingly, even in the presence of Vpu, NK cells expressing iNKRs for HLA-C and HLA-E were able to degranulate albeit two-fold less than NK cells not expressing iNKRs for HLA-C and HLA-E [118].

## 7. Conclusion

**Figure 5:**
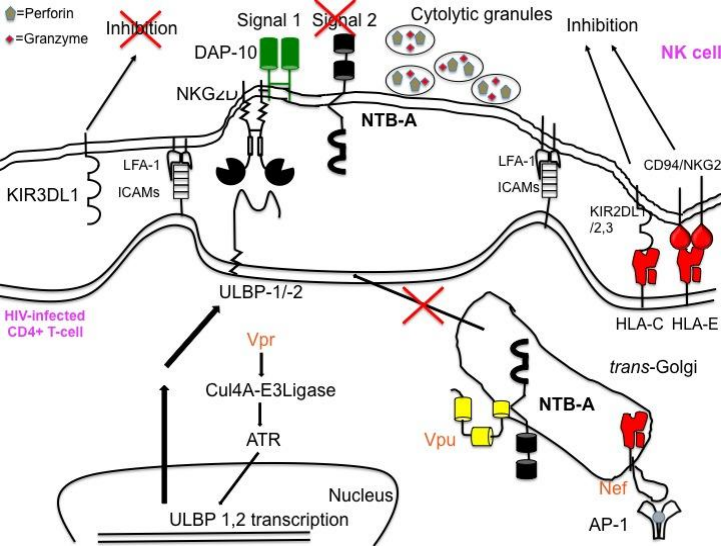
HIV-1 Vpu’s down modulation of NTB-A prevents NK cells from efficiently killing infected cells. Though Vpr induces expression of ligands for the activating receptor NKG2D and Nef down modulates ligands for the NK cell inhibitory receptor, NK cells are still unable to efficiently lyse HIV infected CD4+ T-cells. Vpu’s sequestration of NTB-A in the *trans*-Golgi network in the infected cell prevents NK cells from obtaining a second activating signal to initiate NK cell degranulation.

Our recent studies indicate that HIV-1 evades NK cells. It would seem likely that NK cells would be skewed toward killing infected cells since HIV-1 infection leads to increased expression of ICAMs on the infected cell surface [133], induces expression of ULBP-1 and ULBP-2, and down modulates HLA-A and HLA-B. However, Vpu’s down modulation of NTB-A and the retention of HLA-C and -E prevent efficient degranulation of NK cells (Figure 5). Future work needs to be conducted to better understand the mechanism through which Vpu down modulates NTB-A, and furthermore, why NTB-A, but not other co-activating ligands, is crucial for NK cell lysis of HIV-1-infected cells.

## Figures and Tables

**Table 1:** Natural Killer Cell Activation Receptors.

**Name**	**Ligand**	**Adaptor Protein**
NKp30	Unknown	FcɛRIγ□CD3ζ
NKp44	Unknown	DAP12
NKp46	Unknown	FcɛRIγ□CD3ζ
CD158h/KIR2DS1	HLA-C	DAP12
CD158i/KIR2DS4	HLA-Cw4	DAP12
CD158e2/KIR3DS1	Unknown	DAP12
CD158j/KIR2DS2	Unknown	DAP12
NKG2D	ULBP-1-6, MIC-A,-B	DAP10
CD16	IgG	FcɛRIγ□CD3ζ

Modified from [25].

**Table 2:** Natural Killer Cell co-Activation Receptors.

**Name**	**Ligand**	**Adaptor Protein**
NTB-A	NTB-A	SAP/EAT-2
DNAM-1	CD155/CD112	Protein Kinase C
CRACC	CRACC	EAT-2
2B4	CD48	SAP

Modified from [25].
